# Identification of a Recurrence Signature and Validation of Cell Infiltration Level of Thyroid Cancer Microenvironment

**DOI:** 10.3389/fendo.2020.00467

**Published:** 2020-07-23

**Authors:** Liang Zhang, Ying Wang, Xiaobo Li, Yang Wang, Kaile Wu, Jing Wu, Yehai Liu

**Affiliations:** ^1^Department of Otorhinolaryngology, Head & Neck Surgery, The First Affiliated Hospital of Anhui Medical University, Hefei, China; ^2^Department of Otorhinolaryngology, Head & Neck Surgery, The Second Affiliated Hospital of Anhui Medical University, Hefei, China

**Keywords:** thyroid cancer, signature, GEO, TCGA, recurrence

## Abstract

Though many patients with thyroid cancer may be indolent, there are still about 50% lymph node metastases and 20% the recurrence rates. There is still no ideal method to predict its relapse. In this study, we analyzed the gene transcriptome profiles of eight Gene Expression Omnibus (GEO), and next screened 77 commonly differential expressed genes. Next, Least Absolute Shrinkage and Selection Operator (LASSO) regression model was performed and seven genes (i.e., FN1, PKIA, TMEM47, FXYD6, SDC2, CD44, and GGCT) were then identified, which is highly associated with recurrence data from the Cancer Genome Atlas (TCGA) database. These patients were then divided into low and high-risk groups with specific risk-score formula. Univariate and multivariate Cox regression further revealed that the 7-mRNA signature plays a functional causative role independent of clinicopathological characteristics. The 7-mRNA-signature integrated nomogram showed better discrimination, and decision curve analysis demonstrated that it is clinically useful. Besides, patient with lower risk score shows a relatively lower level of activated dendritic cells (DCs), resting DCs, regulatory T cells and γδT cells, and process of DCs apoptotic. In conclusion, our present immune-related classifier could produce a potential tool for predicting early-relapse.

## Introduction

Thyroid cancer ranks first place in endocrine tumor, and current treatment for papillary thyroid cancer (PTC) are surgery, radioactive iodine ablation, and thyroid hormone replacement (1, 2). Although thyroid cancer usually has a good prognosis, some can still differentiate into more aggressive tumors (3). The 5-years survival rate of stage IV patients is only 50%, and the recurrence rate is as high as 20–30% (4). The cause of recurrence is related to various factors: older patients, larger tumors, cervical lymph node metastasis, extrathyroidal dilation, and lymphatic infiltration (5).

Subsequent recurrence of PTC can lead to surgical trauma, making patients at higher risk for recurrent laryngeal nerve injury (6). These patients with aggressive tissue subtypes and high risk of relapse need radioiodine (I^131^) residual ablation (7). Although thyroid-stimulating hormone (TSH) inhibition is routinely performed after surgery to decrease tumor relapse and improve outcomes, long-term hyperthyroidism may result in various side effects such as atrial fibrillation, osteoporosis, and heart diseases (8). Therefore, the prognosis of PTC is crucial for patients. The incidence of local recurrence and distant metastasis of thyroid cancer is 3 and 1%, respectively (9). But how to distinguish between inert and invasive thyroid cancer is still challenging. In recent years, the molecular basis of the pathogenesis of thyroid cancer has been rapidly recognized (10). Genetic mutations (i.e., BRAF and RAS) are related to pathogenesis and outcome of thyroid cancer (11). In modern treatment, personalized treatment based on the outcome of patients who have thyroid cancer is essential.

It has been well-demonstrated that tumor microenvironment (TME) and tumor cells are close associated, especially the multiplex interaction between cancer cells in TME and infiltration cells is responsible for all stages of carcinogenesis, from the early stage to progression or metastasis (12). TME can regulate the host immune system by changing the composition of immune cells, including recruiting suppressive immune cell fractions, down-regulating neoantigen recognition, level of immunosuppressant ligands, and cancer immune editing (13). Experimental evidence shows that a large scale of chemokines support an immune-cell attracting and maintaining a role in thyroid cancer, enhancing tumor proliferation, angiogenesis, and metastasization (14). Immunotherapy targeting chemokine receptors could reduce the tumor-related inflammation, altering the immune phenotype of the cells infiltrating of the thyroid tumor microenvironment, and ultimately the patient's outcome (15). Tumor recurrence and metastasis hinder the progress of clinical management (16). However, the accuracy of predicting the risk of recurrence with scoring systems is limited. Nowadays, many mRNA signatures were established in predicting prognosis, which provides clinically meaningful and robust tool combining a group of genes with a unique characteristic pattern that occurs in tumor cells.

In our study, we based our effort on eight eligible GEO datasets to identify commonly differentially expressed genes (DEGs), and these DEGs were included in univariate Cox survival and lasso Cox regression models from datasets for thyroid carcinoma of the Cancer Genome Atlas Thyroid Cancer (TCGA-THCA) expression. With clinical profiles, we aim to generate mRNA signature related to progression-free survival (PFS) of PTC. In addition, gene expression profiling was used to enrich the integrated immune cell types (17). Through comprehensive analysis may help to explore the clinical application of the immune landscape to stratify clinical prognosis and the potential significance of biomarkers in the immune microenvironment. Then we used multivariate Cox survival analysis to verify these independent factors. Clinicopathologic nomograms integrated with or without this signature-based stratification were then constructed. Finally, the 7-mRNA signature-integrated nomogram could offer a more accurate prediction of thyroid cancer prognosis than the simply clinicopathologic nomogram. In general, our model with a nomogram might provide an immune-related tool for post-surgery surveillance.

## Methods

### Data Acquisition and Differentially Expressed Gene (DEG) Analysis

We gained raw data (HTSeq-counts and HTSeq-FPKM) and clinical characteristics of TCGA-THCA (https://portal.gdc.cancer.gov/). A total of 568 samples (510 tumor and 58 normal tissues) were enrolled in this study. And the GEO database (http://www.ncbi.nlm.nih.gov/geo/) (GSE29265, GSE33630, GSE3467, GSE3678, GSE5634, GSE58545, GSE60542, and GSE65144) were also collected. Screen for collection criteria: [1] raw transcriptional expression profile with clinical data (only available in TCGA), [2] using the mean value once there is more than one probe for a gene; exclusion criteria: <200 DEGs or including no non-tumor tissues. We define the period from initial treatment to the events (new neoplasm, imaging evidence of disease, or disease recurrence) as PFS. All patients were staged using the thyroid cancer staging system released by the American Joint Committee on Cancer (AJCC) used different tumor-node-metastasis (TNM) classification (18). For these individuals who have no tumor relapse, we use the last follow-up time without a recurrence event. Then, we analyzed thyroid cancer samples compared with normal samples using the R package “Limma” and identified DEGs, which has an adjusted *p*-value (<0.05) and an absolute log_2_(fold change) (log_2_FC) (>0.5).

### Bioinformatics Analysis

Principal component analysis (PCA) can lessen the dimension of the dataset, so that the shared and unique expression patterns can be identified by describing the dataset and its differences. K-Means clustering was also performed. Gene set enrichment analysis (GSEA) was conducted with the foldchange genes and helps to identify activation or inhibition of candidate pathways (19). Gene ontology (GO) and Kyoto Protocol Encyclopedia of Genes and Genomes (KEGG) were analyzed (20, 21), and the genes were divided into different hierarchical categories according to their biological processes and signaling pathways. The enrichment *p* < 0.05 are consider significantly.

### Infiltration of Cells, LASSO Regression Model and Relapse Survival Analysis

We quantified the heterogeneous cellular landscape by Xcell tool (https://xcell.ucsf.edu/), which can get inferring cell types based on transcriptome profiles (22) and gene expression data from TCGA-THCA. Besides, the CIBERSORT algorithm (https://cibersort.stanford.edu/), which uses the 1,000 permutations and LM22 signature (23), was employed to calculate the infiltration of immune cells in thyroid tumors. *P* < 0.05 of the inferred immune cell population was considered accurate. For these screened DEGs, we constructed the LASSO regression models to analyze survival profiles with R package “glmnet,” which can present actively associated variables in higher dimensionality (24, 25). By adopting the function of cv.glmnet, we next selected the best model based on 10-fold cross-validation. While cv.glmnet could generate variable slightly between various times. Thus, we ran up to 100 times of the cv.glmnet and the lambda, and cross-validation errors were averaged.

### Nomogram Evaluation

By using the “rms” package, independent prognostic and pertinent clinical characteristic were added to establish the nomogram to predict 1- and 2- years of PFS and drew calibration plots to validate the nomograms' performance. Concordance index (C index) tested the equilibrium between observed prognostics and predicted events. And we use the decision curve to evaluate its clinical value.

### Statistical Analysis

The optimum cutoff of the risk score was defined for predicting PFS. By the Kaplan-Meier analysis and the log-rank tests, we analyzed the receiver operating characteristic (ROC) curve obtained optimum for predicting survival curves with this cutoff. Univariable and multivariable Cox regression were applied to check the independent factor among clinicopathologic characteristics and multi-mRNA signature. *p*-values, which < 0.05, were regarded as significant.

## Results

### Screen Commonly Genetic Alterations in Thyroid Carcinoma

**Figure 1:**
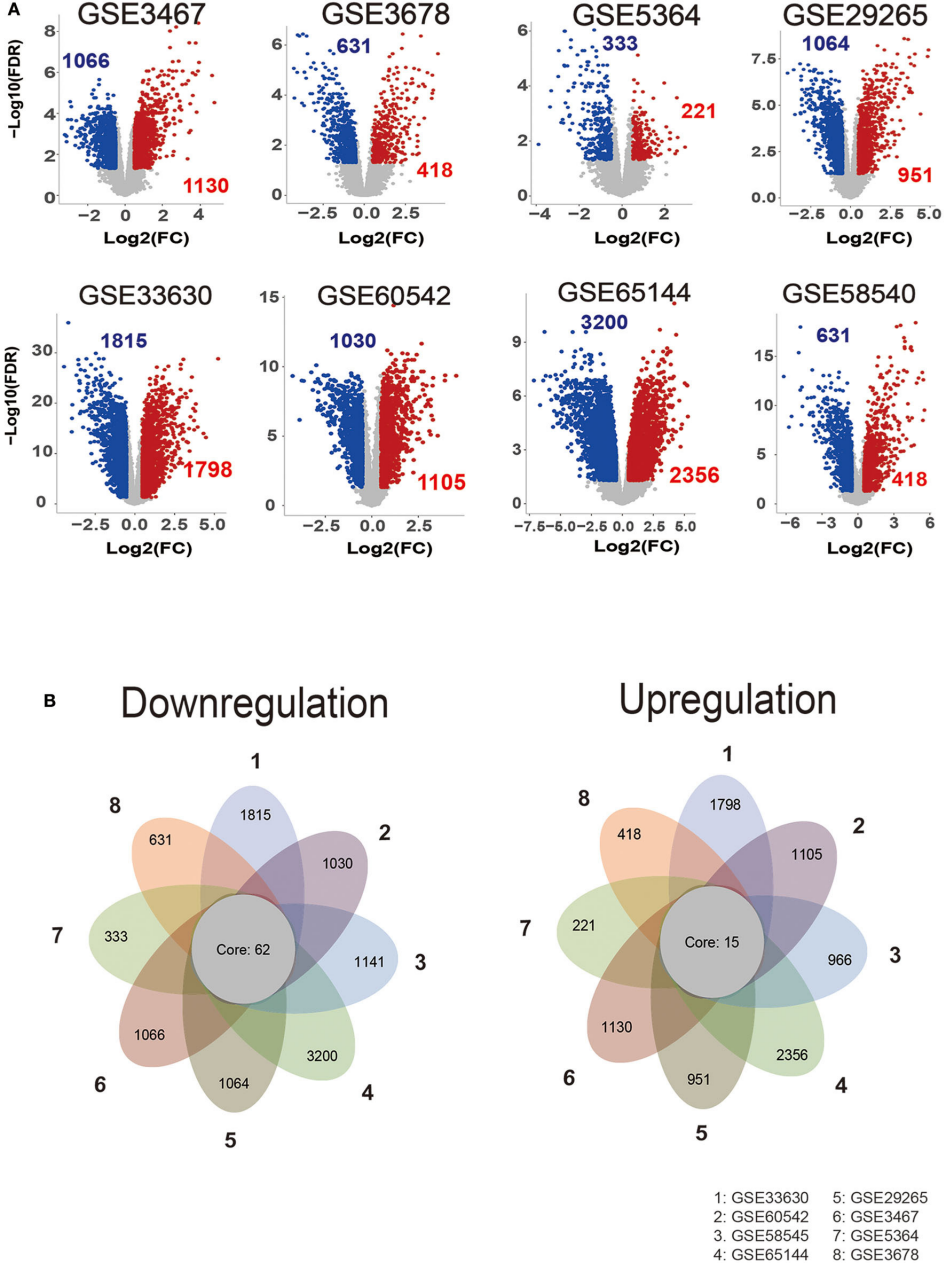
Identification of commonly dysregulated genes in different datasets. **(A)** Volcano plot of DEGs in selected gene expression datasets. **(B)** Flower plot of eight GEO profiles in downregulation and upregulation.

The GEO is a gene expression database that can be analyzed to explore new strategies, such as disease stratification, biomarker identification, and phenotypic comparison, etc. (26). Since the heterogeneity of human samples or experimental platforms, we are supposed to combine analyses with various datasets. Here, our analyses included eight thyroid carcinoma GEO datasets, each containing eight gene sets. According to our screen criteria (fold change > 1.5 and FDR < 0.05), we analyzed DEGs between thyroid cancer group (212 cases) and normal tissue group (160 cases), totally raw data was from the GEO database (listed in Table S1). By using the “Limma” package, we depicted the volcano plots of DEGs (Figure 1A) and performed overlapping analyses to identify the commonly dysregulated gene set in thyroid cancer. Finally, we got 77 DEGs, 15 of which upregulated and 62 downregulated (Figure 1B).

### Consensus-Cluster Analysis Demonstrated Cluster 1 Might Associate With the Recurrence of Thyroid Cancer

**Figure 2:**
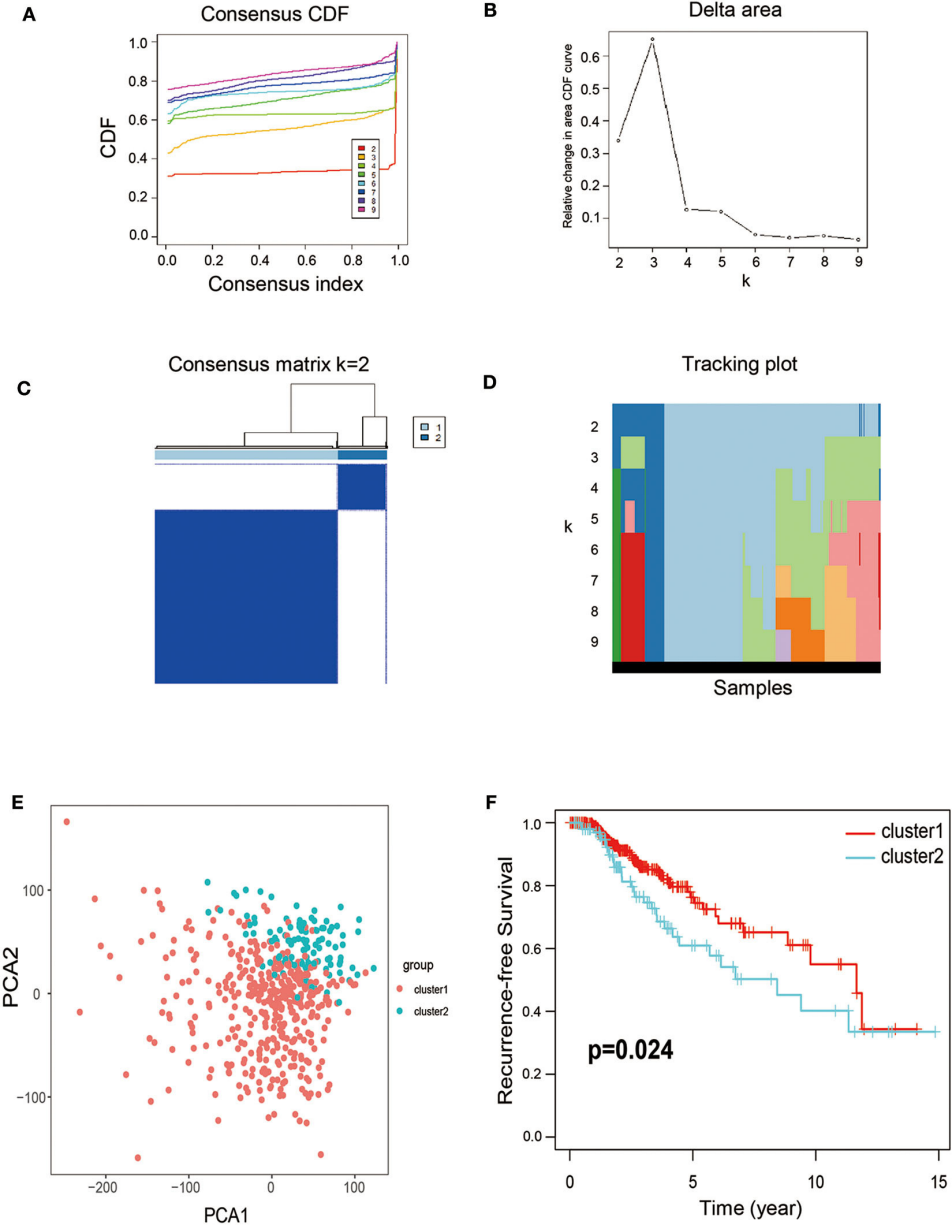
Calculation of consensus clusters by 77 DEGs. **(A)** Consensus clustering cumulative distribution function (CDF) of k = 2–9. **(B)** Relative change in area under CDF curve of K = 2–9. **(C)** Consensus clustering matrix of k = 2. **(D)** The tracking plot for k = 2–10. **(E)** Principal component analysis (PCA) of the overall RNA expression data from TCGA-THCA. **(F)** Recurrence-free survival between the two clusters from TCGA-THCA (*p* = 0.024).

Considering that the 77 DEGs may exert its regulatory role on thyroid cancer synergistically, the correlation and subgroup analysis among DEGs' expression were then performed. As shown in Figure 2A, we used Consensus-Cluster analysis to classify these cancer tissues based on the expression of the 77 DEGs. Nevertheless, the CDF curve demonstrated that k = 2 was preferable as well (Figures 2A,B). As shown in Figure 2C, k = 2 was a reasonable choice, which has cluster stability rising from k = 2 to 10 in PTC dataset. And the tracking plot for k = 2 to k = 10 is shown in Figure 2D. Then, PCA analysis was used to explore the characteristic of expression data based on the stratification of both k = 2 in patients of TCGA-THCA. According to the cluster stratification, our data manifested an obvious difference between these samples in the 2D plotting of PCA data (Figure 2E). Intriguingly, based on this sample cluster method, we found that the PFS between two clusters (*p* = 0.024) (Figure 2F).

### Identification and Evaluation of a 7-Gene Immune-Related Signature From TCGA-THCA

**Figure 3:**
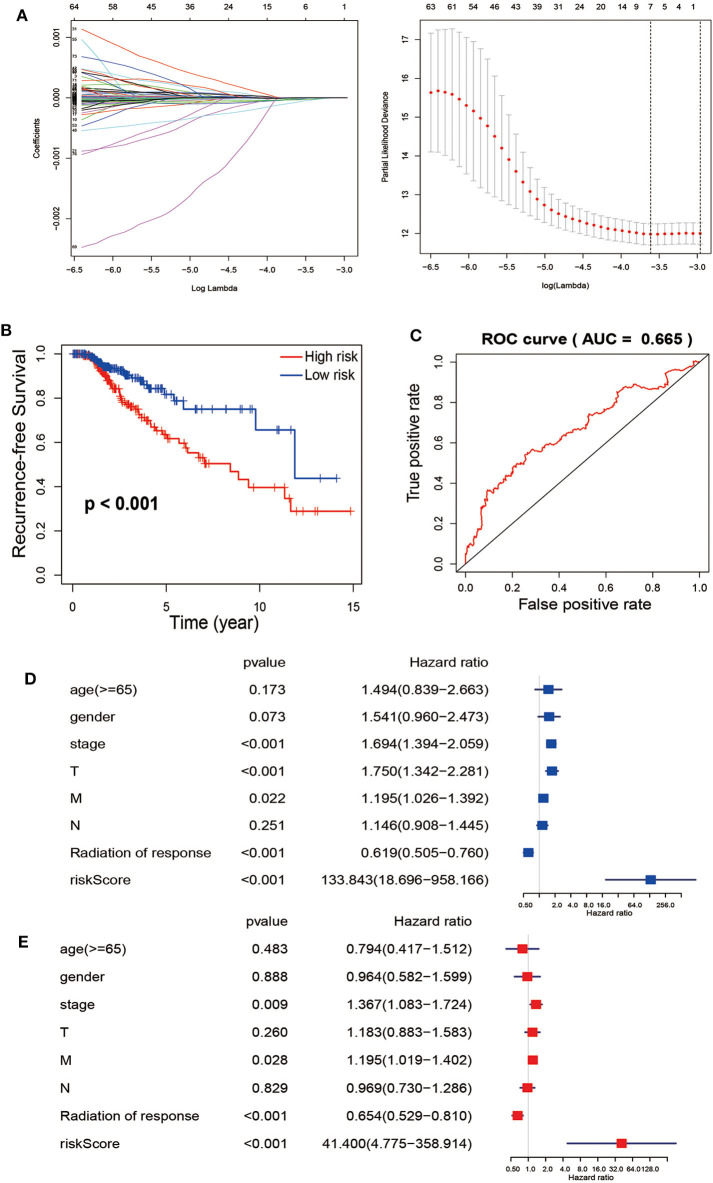
The Cox regression analysis for estimating the clinical value of the risk score. **(A)** For construction of a signature from TCGA-TCHA cohort, Twelve-fold cross-validation, where optimal λ lead to 24 non-zero coefficients, is applied for selecting tuning parameters in the LASSO model. The best values by minimum- (lambda.min, left vertical dotted line) and 1-SE- criteria (lambda.1se, right vertical dotted line) representing the dotted vertical lines. Partial LASSO coefficient profiles of the 77 mRNAs. **(B)** Recurrence-free survival between patients with high risk (red) and low-risk (blue). **(C)** Calculate the area under the ROC curve for risk score. The univariate **(D)** and multivariate **(E)** Cox regression analysis of age, gender, grade, TNM stage, radiation of response and risk score, age, gender, pathological stage, and TNM stage of the whole survival risk score according to the ROC curve.

For avoiding overfitting and reducing the feature dimension, we employed the LASSO regression model on these mRNAs based on the PFS trait of thyroid cancer samples. We generated 7 mRNAs by multiple Cox regression analyses, including FN1, PKIA, TMEM47, FXYD6, SDC2, CD44 and GGCT were further identified from the above 7 mRNAs. Thus, we can conclude that the expression profiles in tumors showed that in the low-risk group, PKIA, TMEM47, FXYD6, SDC2, and GGCT were highly expressed, while FN1 and CD44 were expressing in the opposite direction (Figure 3A). Based on the expression of these mRNAs, the risk score was calculated for each patient. Risk score = FN1 × 1.86e-08—PKIA × 3.43e-05 −TMEM47 × 7.30e-05−FXYD6 × 2.63e-05−SDC2 × 1.24e-05 + CD44 × 9.05e-07−GGCT × 1.19e-05. According to the median score, thyroid cancer samples were divided into low- and high-risk subgroups. It is showed that these patients in the high-risk group tend to have a high-frequency PFS than the low-risk group via Kaplan-Meier curve, suggesting the signature of risk score is applicable (*p* < 0.001) (Figure 3B). Collectively, these studies identified 7-immune related DEGs could act as causatively predictive signature for PTC. In addition, ROC analysis was used to check the specificity and sensitivity of the calculative role on the causative prediction of patients with tumor, and the area under the ROC curve was 0.665 (Figure 3C). Next, we performed the univariate and multivariate Cox regression analyses to investigate the 7-mRNA signature and clinicopathological factors (age, gender, pathological stage, T, N, M, and radiation of response) were independent factors for predicting relapse. The hazard ratio of risk score and 95% Confidence Interval were 133.843 and 18.696–958.166 in univariate Cox regression analysis (*p* < 0.001) (Figure 3D), 41.4 and 4.755–358.914 in multivariate Cox regression analysis (*p* < 0.001) (Figure 3E), respectively. To sum up, our data suggest that the 7 immune-related mRNAs might act as an independent indicator for predicting outcome of PTC patients.

### Establishment of a Clinical Nomogram Based on 7-mRNA Signature

**Figure 4:**
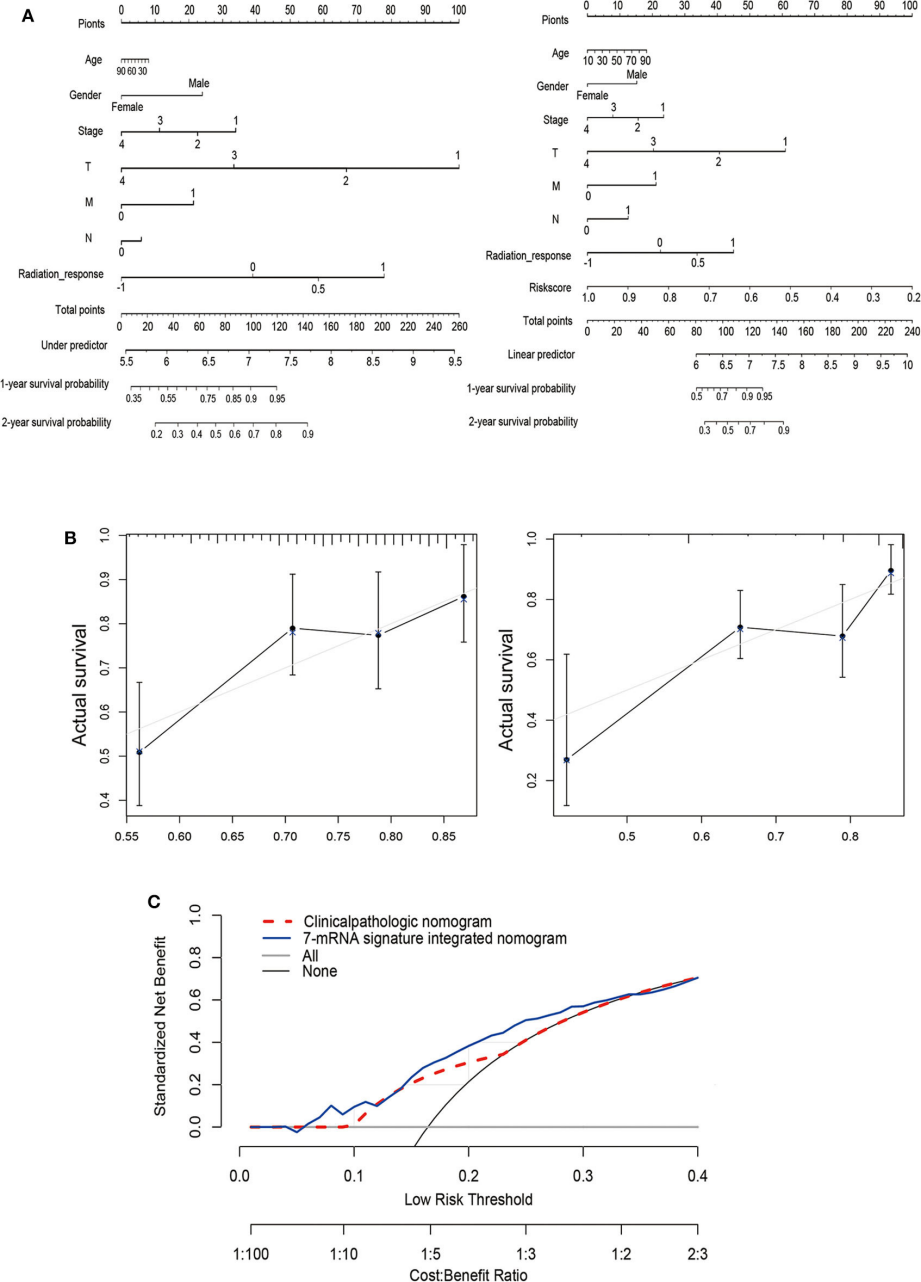
Establishment of a nomogram based on clinical characteristics and the 7-mRNA-gene signature. **(A)** Nomograms combined without (left) or with (right) the 7-mRNA-gene signature to predict 1- and 2-year PFS probability in THCA. **(B)** Calibration curves for the clinicopathologic nomogram and the 7-mRNA-signature integrated nomogram calibrate each model based on the consistency between the predicted and observed results. **(C)** Decision curve analysis of this nomogram.

To generate clinically applicable tools to predict PFS of thyroid cancer, we established a nomogram that integrates 7-mRNA signatures and multiple clinical-pathological risk variables such as gender, age, pathological stage, and T, N, M, of which were determined to be associated with PFS by univariate Cox regression analysis (left), and a simple clinicopathological nomogram integrated with 7-mRNA signature (right) is shown in Figure 4A. For these nomograms, we constructed calibration curves for the recurrence after surgery is shown in Figure 4B (left and right, respectively). Similarly, the integrated line chart of 7-mRNA signature shows better consistency with the actual observation results than the clinicopathological line chart (C index of the clinicopathological nomogram: 0.244, 95% CI: −0.513–0.513; C index of 7-mRNA integral nomogram: 0.349, 95% CI: −0.303–0.303; *P* < 0.001). In addition, a nomogram is calculated by a decision curve analysis (Figure 4C). The results show that the overall net benefit of the 7-mRNA signature integration nomogram is higher than the clinicopathological nomogram.

### Classification Analysis of the 7-mRNA Signature

**Figure 5:**
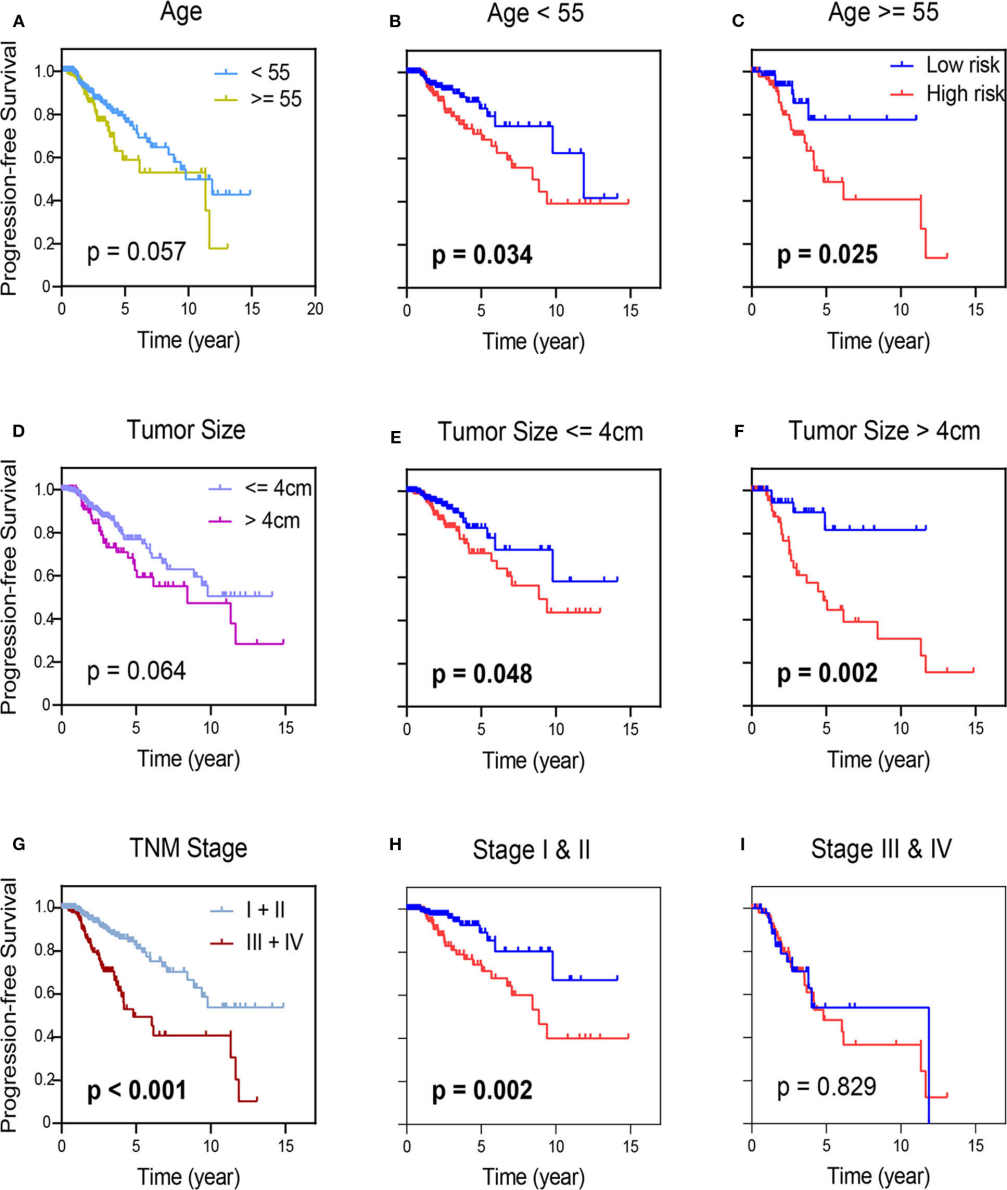
Kaplan-Meier survival analysis in groups stratified by clinical indexes, age **(A)**, tumor size **(D)**, and TNM staging **(G)**. Kaplan-Meier survival analysis according to the 7-mRNA-based signature in these groups with age (<55) and age (≥55) in TCGA-THCA **(B,C)**, and in subgroups of patients with tumor size (≤ 4 cm) and age (>4 cm) in TCGA-THCA **(E,F)**, in subclasses with TNM stage I & II and III & IV in TCGA-THCA **(H,I)**.

In order to determine prognostic utility of the 7-mRNA based signature for the most widely used AJCC staging system can predict outcomes of patients (27), we further applied survival analysis concerning the 7-mRNA-based signature in these subgroups with different clinical parameters (Figure 5). For the TCGA-THCA cohort, when subdivided by these clinical parameters (age, tumor size, and pathologic staging), the 7-mRNA-based signature was still playing a statistically significant prognostic role for PFS. As expected, these with older age, larger tumor size, and more advanced TMN stage tend to have a worse prognosis (Figure 5). Interestingly, Kaplan-Meier analysis of these groups divided by age or tumor size shows no statistically significant (Figures 5A,D). Promisingly, when classified by 7-mRNA signature, these who manifest a high-risk score have a worse prognosis in either young (<55) or older (> = 55) group (Figures 5B,C). Similarly, 7-mRNA classifier could further strengthen tumor size stratum (Figures 5E,F). Nevertheless, the PTC patients in TCGA with lower risk scores are also better PFS in early TNM stages. Thus, the 7-mRNA-based signature could strengthen the prognostic ability of the early-TNM staging (Figures 5G–I).

### The Correlation of the Risk for Tumor Recurrence and Cell Infiltration Landscape

**Figure 6:**
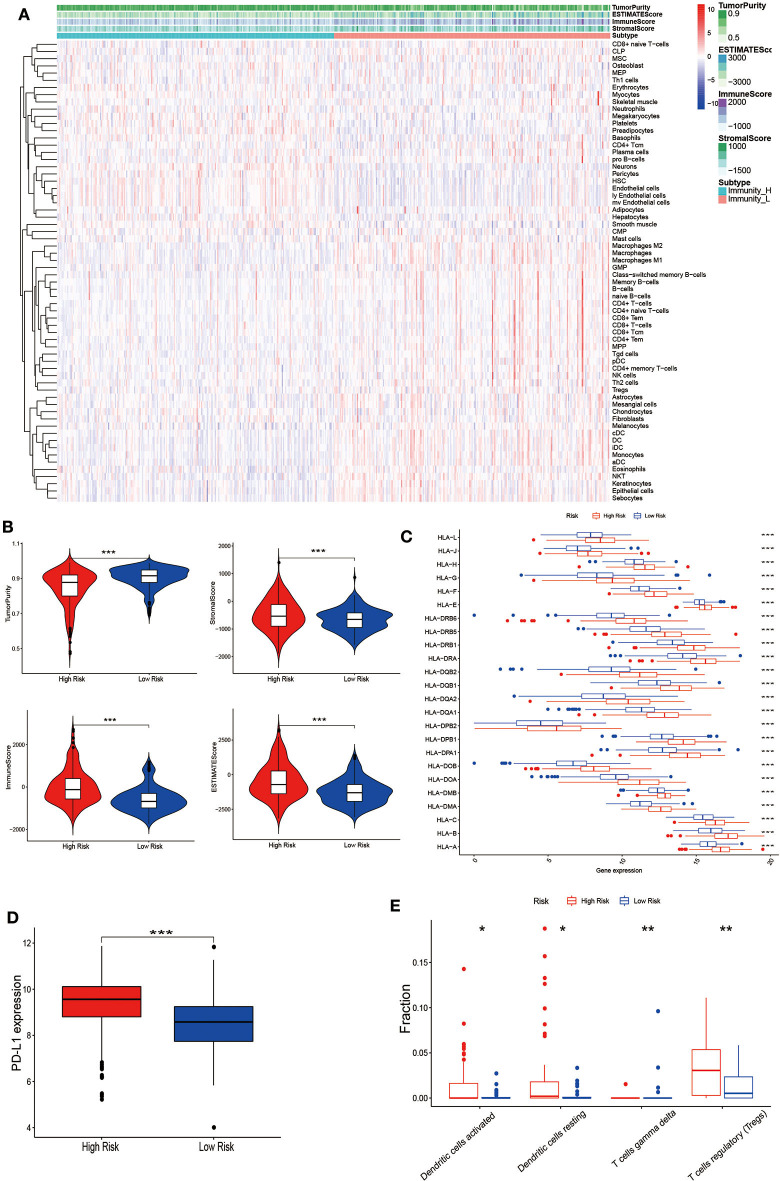
Establishment and verification of thyroid cancer grouping. **(A)** Expression of infiltrating cells in Low-risk and High-risk groups, representing the opposite infiltrate degree via the Xcell tool. **(B)** Tumor Purity, Stromal Score, Immune Score and ESTIMATE Score of patients with the grouping information were showed; the difference of the four-index between these two groups (*p* < 0.001). **(C)** The level of HLA family genes and **(D)** PD-L1 in the high-risk group (red) were all significantly higher than those in the low-risk group (blue) (*p* < 0.001). **(E)** The difference of each immune cell in two groups by CIBERSORT analysis. (*p < 0.05, **p < 0.01, ***p < 0.001).

Gene expression data from TCGA-THCA was collected to quantify the cell infiltrate fraction in tumor tissues and Xcell tool was performed to the expression profile of thyroid cancer tissues to compute the infiltrate fraction of cells. We finally got 64 risk related cells (Fibroblasts, Neurons, Preadipocytes, Platelets, Hepatocytes, NKT, Epithelial cells, Basophils, Tregs, Keratinocytes, CD4^+^ T-cells, Eosinophils, CD8^+^ T-cells, HSC, CD4^+^ memory T-cells, Adipocytes, Melanocytes, CD8^+^ Tcm, Sebocytes, CD4^+^ naïve T-cells, NK cells, CLP, Naïve B-cells, CD4^+^ Tcm, Neutrophils, Th2 cells, Megakaryocytes, Smooth muscle, MPP, GMP, Plasma cells, pro B-cells, CD8^+^ naïve T-cells, ly Endothelial cells, iDC, Astrocytes, Tgd cells, Macrophages M2, B-cells, DC, Monocytes, pDC, CD8^+^ Tem, Memory B-cells, Macrophages, MSC, Macrophages M1, Skeletal muscle, Endothelial cells, Mesangial cells, mv Endothelial cells, Myocytes, aDC, CD4^+^ Tem) (Figure 6A). These immune-related terms were involved to eradicate the richness of different cells. Through the supervised hierarchical risk clustering method, thyroid cancer samples were stratified into the high infiltration group (*n* = 243) and low-risk group (*n* = 244). To investigate the availability of the grouping category, we used the ESTIMATE algorithm to estimate Stromal Score, ESTIMATE Score, Immune Score, and Tumor Purity (Figure 6B). The diagrams show that the group with high immune cell infiltration has a significantly higher level of ESTIMATE Score, Immune Score and the Stromal Score. However, immune infiltration and Tumor Purity are negatively correlated. That is, the high-risk group had higher immune components but lower tumor purity (*p* < 0.001). Furthermore, the level of HLA family and PD-L1 were significantly higher in the high-risk group (*p* < 0.001) (Figures 6C,D). In the light of these 7-mRNAs that were associated with tumor immunity, the correlation between the signature and the infiltration of immune cell subtypes in thyroid cancer were further analyzed by using the LM22 reference. Among them, activated DCs, resting DCs, regulatory T cells and γδT cells were associated with poor prognosis (Figure 6E), indicating the infiltration of these cell types was positively relevant to the relapse.

### GSEA and GO Analysis of Risk-Dependent Groups

**Figure 7:**
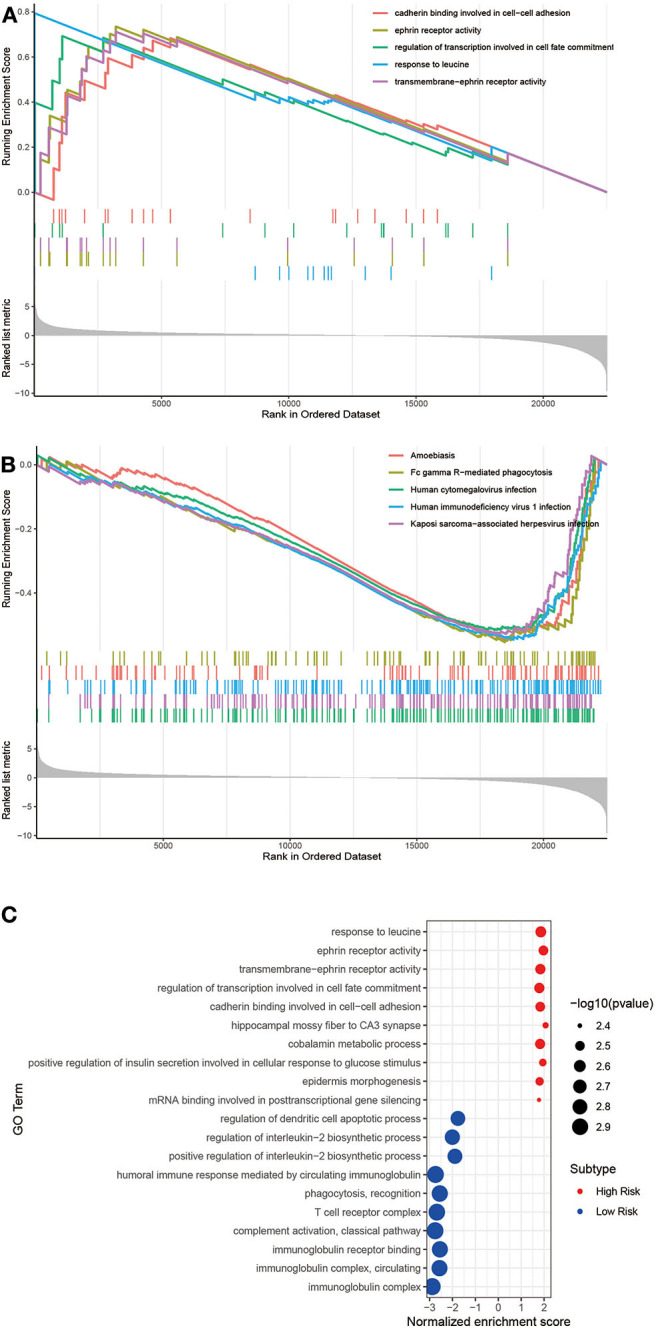
Gene Set Enrichment Analysis (GSEA) and GO analysis of these two groups. **(A)** The significant GSEA analysis results of High risk and **(B)** Low risk. The total GSEA data of clusters were listed in Tables S2, S3. **(C)** Bubble plot of GO analysis.

To understand the function of this signature, these dysregulated genes were submitted to Gene Set Enrichment Analysis (GSEA) using GO and KEGG pathway enrichment analyses, separately. The top 5 upregulated GO analysis in Biological Processes, Molecular Function, and Cellular Component (Figure 7A) were listed. Meanwhile, we have enriched five KEGG pathways by downregulated genes (Figure 7B). Finally, as shown in the bubble figure (Figure 7C), by using GO analysis, most enriched in regulation of DCs apoptotic process, regulation of interleukin-2 biosynthetic process and positive regulation of interleukin-2 biosynthetic process in the upregulated genes. Noteworthy, enrichment pathway analysis showed that a higher expression of the signal pathways of DCs apoptosis and T cell receptor complex in the low-risk group. Contrarily, terms such as response to leucine, ephrin receptor activity and transmembrane-ephrin receptor activity were enriched by downregulated genes.

## Discussion

Thyroid cancer is one of the most common cancers. Though it usually shows a good prognosis, many patients still suffer recurrence after treatment (28). The prognostic tools such as MACIS scoring, and AJCC staging system only focus on clinical characteristics and difficult to accurately estimate a specific risk of relapse for individual patients (29–31). In the light of thyroid cancer tissue contain malignant and other normal cells, including immune and stromal cells (32). These cells evolve together, form a complex whole, communicate with each other to form complex signaling (33, 34). With the development of high-throughput technology and the maturate mechanism of public data, unparallel and extensive multiple tumor data stocked up in the available database. Using the transcriptome sequencing data, mRNAs, and clinical characteristics of thyroid cancer gained from TCGA-THCA, we established the 7-mRNA immune-infiltration related prognostic signature. Also, we concentrate on the heterogeneity of thyroid cancer and the association between tumor cells and tumor-infiltrating immune cells, providing a promising method for studying carcinogenesis and mining new diagnostic and therapeutic meaning. Although the current prognosis tools for PTC has been explored, integrating multiple biomarkers into one model could improve the current guide and provide a more accurate decision for clinicians independent of age, tumor size, and early-staging. In this study, we found that these seven genes are closely related to immune cells, activated DCs, resting DCs, regulatory T cells and γδT cells were associated with the risk of recurrence. Furthermore, the results of the enrichment analysis showed that the signal pathways of DCs apoptosis and T cell receptor complex have a higher expression in the low-risk group.

To verify the clinical available of the gene signature, we matched it with the clinical characteristics of PTC patients, including gender, age, TNM stage, response of radiation, etc. Through the univariate, multivariate COX analyses, and ROC analysis, we demonstrated the 7-mRNA prognostic signature could be an independent prognostic factor in PTC patients. Now, we identify 7 mRNAs including PKIA, TMEM47, SDC2, CD44, GGCT, FXYD6, and FN1 as prognostic signatures for PTC. To the best of our knowledge, the roles of PIKA, TMEM47, SDC2, and GGCT are not reported to be involved in thyroid cancer yet. Satarupa et al. point out that PIKA is an essential biomarker for cervical cancer staging (35). TMEM47 is significantly associated with the biological processes of aggressive breast cancer (36). SDC2 could regulate cell proliferation and recruit immune cells (37), and the level of SDC2 methylation in fecal DNA could be a diagnostic method for early colonel rectal cancer (38). CD44 is an unfavorable factor for overall survival and disease-free survival in medullary thyroid carcinoma (39). Keiko et al. show that loss of GGCT leads to autophagy in breast and prostate cancer cells *in vitro* (40). FXYD6 is also a new biomarker to predict the progression-free interval of PTC (41), and FN1 was positively related to the pathologic stage of PTC patients (42).

The immune microenvironment in thyroid cancer is extremely heterogeneous, and there is a greatly various type and quantity of immune cells. And Tumor Purity, Immune Score, ESTIMATE Score, and Stromal Score are statistically significant in between the high- and the low-risk cell-infiltration group. Based on mRNA profile, tumor immune infiltrating cells occupies a high fraction in various cancer. In present studies, activated DCs, resting DCs, regulatory T cells, and γδT cells were associated with poor prognosis. It has indicated that the presence of tumor-infiltrating DCs is related to tumor progression and prognosis. DCs in tumors have the effect of antigen presentation and activation, directly activating T cells (43). T cells have updated continuously functions in the body and can exist in different dynamic stages or functional subclasses simultaneously (44). For immune response, different subgroups of T cells have different functions, such as releasing lymphokines, killing target cells, assisting immune response and memory-specific antigen stimulation (45). γδT cells account for a small portion of the whole T lymphocytes (0.5–5%), and their tissue distribution is variable. These cells are the main line of defense against pathogens that invade early in life. It is recognized that tumor-associated DCs infiltration can promote tumor progression through tumorigenesis, angiogenesis, and disruption of the adaptive immune response (46). T cell penetration is attributed to promote malignant cell migration and metastasis through the androgen- and estrogen- receptor signaling (7). Enrichment pathway analysis showed that the signal pathways of DCs apoptosis and T cell receptor complex have a high level in the low-risk group. Apoptosis of DCs can reduce the infiltration of cancer cells after surgery, inhibit cancer recurrence, and improve prognosis. Regions of T cell receptors, which is determining by the complementary of antigen and antibody, is substantial for recognizing tumor—associated antigen. Thus, quantifying T cell receptors could be a supportive tool for diagnosis and therapy in immune infiltrated tumor. Given that a considerable immune infiltrating cells existing in postoperative cancer tissue, we could get the whole tumor immune microenvironment data in the results of RNA sequencing.

Besides, by the expression of HLA and PD-L1 by the algorithm of CIBERSORT, we tested the heterogeneous immune microenvironment in thyroid cancer. The latest success of immune checkpoint blockade therapy with potent and long-lasting responses in various types of solid tumors indicates its meaningful use for refractory, advanced thyroid cancer. At present, the total mutation burden as well as the level of checkpoint inhibitors (such as PD-L1 levels in anti-PD-L1 treatment) have been proposed as predictive indicators of clinical effect (47). Na and Choi observed these immunosuppressive markers (PD-L1, CTLA-4, and HLA-G) is positively associated with the unfavorable relapse-free survival for PTC (12). Our results show that HLA and PD-L1 can be used to predict the risk of recurrence.

Overall, our study found the 7-mRNA signature as prognostic signatures for thyroid cancer. Meanwhile, it is relevant to the infiltration of immune cell subtypes.

## Data Availability Statement

Publicly available datasets were analyzed in this study, these can be found in The Cancer Genome Atlas (https://portal.gdc.cancer.gov/) (TCGA-THCA); the NCBI Gene Expression Omnibus (accession numbers listed in the Supplementary Materials).

## Author Contributions

YL was involved in the entire study project, including study design, experimentation, data analysis, and result interpretation. LZ and YiW completed the data analysis and drafted the manuscript. XL, YaW, KW, and JW contributed to data analysis and manuscript preparation. All authors contributed to manuscript revision and approved the submitted version.

## Conflict of Interest

The authors declare that the research was conducted in the absence of any commercial or financial relationships that could be construed as a potential conflict of interest.
