# Supplementary material for: Identification of a Recurrence Signature and Validation of Cell Infiltration Level of Thyroid Cancer Microenvironment
Source: Front Endocrinol (Lausanne). 2020 Jul 23;11:467. doi: 10.3389/fendo.2020.00467 (PMC7390823; doi:10.3389/fendo.2020.00467)
Supplement: Supplementary file 1 [file Table_1.DOCX]

Table S1 GEO database

| Database | Source | Sample | |
| --- | --- | --- | --- |
|  |  | Tumor | Normal |
| GSE29265 | <https://www.ncbi.nlm.nih.gov/geo/query/acc.cgi?acc=GSE29265> | 29 | 20 |
| GSE33630 | https://www.ncbi.nlm.nih.gov/geo/query/acc.cgi?acc=GSE33630 | 60 | 45 |
| GSE3467 | <https://www.ncbi.nlm.nih.gov/geo/query/acc.cgi?acc=GSE3467> | 9 | 9 |
| GSE3678 | <https://www.ncbi.nlm.nih.gov/geo/query/acc.cgi?acc=GSE3678> | 7 | 7 |
| GSE5634 | <https://www.ncbi.nlm.nih.gov/geo/query/acc.cgi?acc=GSE5364> | 35 | 16 |
| GSE58545 | <https://www.ncbi.nlm.nih.gov/geo/query/acc.cgi?acc=GSE58545> | 27 | 18 |
| GSE60542 | <https://www.ncbi.nlm.nih.gov/geo/query/acc.cgi?acc=GSE60542> | 33 | 32 |
| GSE65144 | <https://www.ncbi.nlm.nih.gov/geo/query/acc.cgi?acc=GSE65144> | 12 | 13 |
